# Long term (1997-2014) spatial and temporal variations in nitrogen in Dongting Lake, China

**DOI:** 10.1371/journal.pone.0170993

**Published:** 2017-02-06

**Authors:** Zebin Tian, Binghui Zheng, Lijing Wang, Liqiang Li, Xing Wang, Hong Li, Stefan Norra

**Affiliations:** 1 College of Water Sciences, Beijing Normal University, Beijing, China; 2 State Environmental Protection Key Laboratory of Drinking Water Source Protection, Chinese Research Academy of Environmental Sciences, Beijing, China; 3 Ecological and Environmental Monitoring Center of Dongting Lake of Hunan, Yueyang, China; 4 Institute of Geography and Geoecology, Karlsruhe Institute of Technology, Karlsruhe, Germany; CAS, CHINA

## Abstract

In order to protect the water quality of Dongting Lake, it is significant to find out its nitrogen pollution characteristics. Using long-term monthly to seasonally data (1997–2014), we investigated the spatial and temporal variations in nitrogen in Dongting Lake, the second largest freshwater lake in China. The average concentrations of total nitrogen (TN) in the eastern, southern, and western parts of the lake were 1.77, 1.56, and 1.35 mg/L, respectively, in 2014. TN pollution was generally worse in the southern area than in the western area. Concentrations showed temporal variation, and were significantly higher during the dry season than during the wet season. Based on the concentration and growth rate of TN, three different stages were identified in the long term lake data, from 1997 to 2002, from 2003 to 2008, and from 2009 to 2014, during which the concentrations and the growth rate ranged from 1.09–1.51 mg/L and 22.09%-40.03%, 1.05–1.57 mg/L and -9.05%-7.74%, and 1.68–2.02 mg/L and 57.99%-60.41%, respectively. The main controls on the lake water TN concentrations were the quality and quantity of the lake inflows, spatial and temporal variations in hydrodynamic conditions within the lake (flow velocity, flow direction), and point and nonpoint inputs from human activities. Diffuse nutrient losses from agricultural land are a significant contributor. As a priority, the local government should aim to control the pollutant inputs from upstream and non-point nutrient losses from land.

## Introduction

Significant disturbances from anthropogenic activities over the past decades have accelerated the process of eutrophication and threatened the health of aquatic ecosystems [1, 2]. Excessive nutrient inputs are the main reason for lake eutrophication [3], and surplus nitrogen (N) is one of the main driving factors [4]. In particular, more than 80% of eutrophic waters in China suffer from serious N pollution [5, 6]. N concentrations distributions are related to the hydrodynamic conditions and anthropogenic input intensity in a river basin [7]. Over the past decade, most studies have identified that different industrial and agricultural activities under different hydrodynamic conditions are the main sources for nitrogen loads [8–12]. However, there is no significant reduction in nitrogen concentration, after the government implemented effective abatement of point sources pollution [12]. The contribution from fertilizer N applications has become a major cause of TN accumulation [13]. Studies showed that China has become the largest consumers of nitrogen fertilizers in the world, which accounted for 35.1% of the world's nitrogen fertilizer application [14]. While the linkage between anthropogenic activities N input and nitrogen concentrations have been poorly documented [15]. Thus, it is important to understand and characterize the sources of human-induced N inputs and their contributions to the N concentrations. Riverine transport is the principal pathway by which point and non-point sources move from land to lake [16]. The upper rivers not only bring in the N loads, but also affect the hydrodynamic conditions of the lake [17], eventually influence the distribution and transportation of nitrogen [18]. However, studies to date rarely provide an integrated analysis of the upper reaches and the lake body [19, 20] and the driving mechanisms for N concentrations distributions associated with upper rivers. Therefore, it is important to reveal the spatial and temporal variations in nitrogen response to the changes of hydrodynamic conditions caused by upper rivers and the N inputs from different sources.

Dongting Lake is located in the middle of the Yangtze River, one of the most polluted river system in China [21]. It is the second largest freshwater wetlands in China, and is the first large seasonal storage lake connected to the Yangtze River for the area downstream of the Three Gorges Dam. The watershed covers an area of about 2.62×10^5^ km^2^ and accounts for about 14% of the entire Yangtze River Basin [22]. The high velocity (0.41 m/s) and the short resident time (20 days) make the transportation and distribution of nutrient very sensitive to the lake hydrodynamic condition changes caused by upstreams [23, 24]. In addition, it is one of the most developed agricultural regions in China with a cultivated area of about 9.7×10^5^ ha that covers 61.29% of the whole basin; indeed, 5.78% of the total grain production of the whole country comes from this area [25]. The intensive anthropogenic activities has increased nitrogen loads and accelerated the eutrophication status [26]. In addition, frequent occurrence of seasonal hydrological droughts caused by the low upstream discharges has resulted in the reduction of water level and the decrease of self-clarification ability in the lake [27], which aggravated the environmental challenges of the lake ecosystem. As such, we choose Dongting Lake basin as the research area and used NANI model; with a focus on nitrogen, and using monthly data for 18 years in Dongting Lake, the main aims of this study were 1) to analyze the temporal and spatial distribution of nitrogen in Dongting Lake and in its upstream waters, 2) to discuss the casual factors for the trends and variations in nitrogen in different lake areas and different time periods because of human activities and hydrological conditions, and 3) to identify the main sources of, and measures to control, nitrogen pollution in Dongting Lake, so as to prevent further eutrophication and to protect the water quality.

## Study area and methods

### Study area

Dongting Lake is located in the middle and lower reaches of the Yangtze River (111°53′–113°05′E; 28°44′–29°35′N) in China, with a water surface area of approximately 2,625 km^2^ and a volume of about 1.78×10^10^ m^3^. The lake has a mean water depth of about 6.39 m and a maximum depth of about 18.67 m during the flood season. The annual average residence time is 20 days, which is rather short compared to Taihu Lake (300 days). Dongting Lake receives recharge from three inlets from the Yangtze River in the northwest, the Songzi, Taiping, and Ouchi water inflows, and four upstream rivers, the Lishui and Yuanjiang rivers in the west, and the Zishui and Xiangjiang rivers in the south. The water discharge of the three water inlets and the four upstream rivers controls 91% of the recharge entering the lake [27]. The water network of the Yangtze River, the upstream rivers, and finally Dongting Lake, mean that there are complex interactions between the Yangtze River and the lake, and also between the upstream rivers and the lake [27].

Generally, the surface area of Dongting Lake can be divided into three parts, namely East Dongting (ED, 1,217 km^2^), South Dongting (SD, 897 km^2^), and West Dongting (WD, 284 km^2^). Because of the combined influence of rainfall around the lake, transit water from the Yangtze River, and water discharge from four upstream rivers in the lake basin, Dongting Lake is characterized by dramatic intra-annual fluctuations in its water level and water storage capacity. Its water storage capacity and water levels in the wet season are about 17–31 times and 1.14–1.64 times, respectively, that of the dry season, which mean that the ecosystem is sensitive to changes in the water level [28]. The water storage capacities of ED, SD, and WD are between 3 and 68×10^8^ m^3^, 2 and 62×10^8^ m^3^, and 1 and 17×10^8^ m^3^, respectively [29]. The water levels of ED, SD, and WD range from 20.21 to 33.28 m, from 19.43 to 31.48 m, and from 28.91 to 32.82 m, respectively.

### Water sampling and data collection

**Fig 1:**
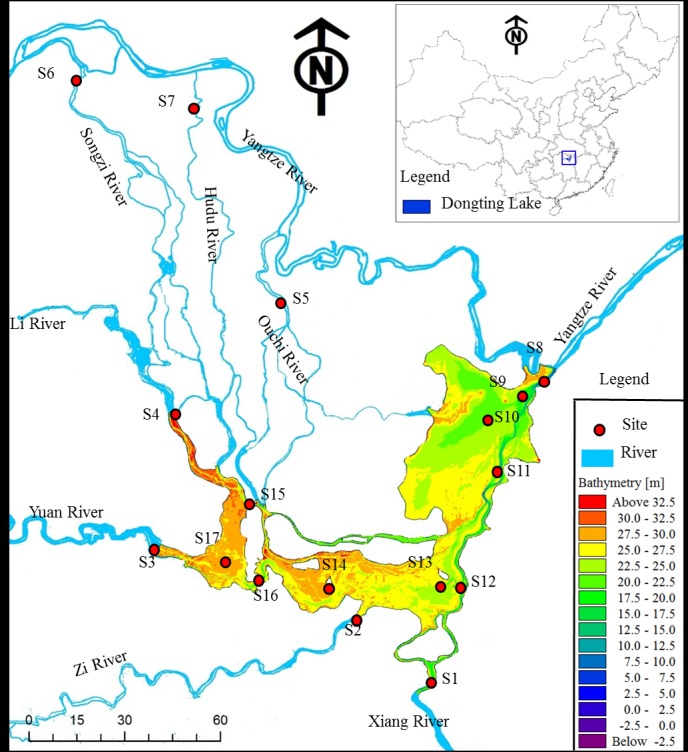
Map of Dongting Lake and the water sampling sites.

Surface water samples were collected from a depth of 0.5 m at 17 sites (S1–S17, Fig 1), and each site had three sampling points (left, middle, right). The samples were divided into four spatial groups that covered the water quality of the entire basin: S1–S7 represented flows from the three water inlets and the four upstream rivers, S8–S11 represented ED, S12–S14 represented SD, and S15–S17 represented WD **(**Table 1). The field study was not carried out on private land and no specific permissions were required for these locations/activities. Endangered or protected species was not involved in the study.

**Table 1:** Information about the sampling locations in Dongting Lake.

Location	Site	Longitude	Latitude	Representation
**Xiangjiang estuary**	S1	112.79	28.56	Upstream river: Xiangjiang
**Zishui estuary**	S2	112.39	28.61	Upstream river: Zishui
**Yuanjiang estuary**	S3	112.10	28.90	Upstream river: Yuanjiang
**Lishui estuary**	S4	112.06	29.27	Upstream river: Lishui
**Ouchi inlet estuary**	S5	112.39	29.59	Yangtze River inlet: Ouchi
**Songzi inlet estuary**	S6	111.79	30.31	Yangtze River inlet: Songzi
**Taiping inlet estuary**	S7	112.12	30.23	Yangtze River inlet: Hudu
**East Dongting (ED)**	S8	113.13	29.43	Outlet of Dongting Lake
	S9	113.07	29.39	Outlet of ED
	S10	112.99	29.32	ED centrol area
	S11	113.00	29.15	Lake entrance from SD to ED
**South Dongting (SD)**	S12	112.88	28.82	Confluence of Xiangjiang and Zishui
	S13	112.85	28.84	SD centrol area
	S14	112.44	28.79	SD centrol area
**West Dongting (WD)**	S15	112.29	29.06	Confluence of Lishui and Songzi
	S16	112.31	28.85	Outlet of WD
	S17	112.20	28.86	WD centrol area

Samples were collected three times each year from 1997 until 2004 (January, May, September), and monthly from January 2005 until December 2014. Total nitrogen (1997–2014), nitrate (2014), and ammonium (2014) were determined by UV spectroscopy after filtration through cellulose acetate membranes (0.45 μm), following the standard methods and guidelines of the Chinese National Quality Standards.

The annual average discharges of S1–S7 were 659.7×10^8^, 229.6×10^8^, 640.6×10^8^, 147.7×10^8^, 326×10^8^, 417×10^8^, and 162×10^8^ m^3^, respectively, based on data collected from the Yangtze River Sediment Bulletin. Information about population, gross domestic product, chemical fertilizer applications, industrial wastewater, domestic sewage, and wastewater treatment throughout the entire basin was collected from the Statistical Yearbooks of China.

### Estimation of Net Anthropogenic N Inputs (NANIs)

N input from human activities in Dongting Lake Basin was estimated from the NANI model introduced by Howarth [30]. NANI is a simple quasi-mass-balance approach, which is the sum of atmospheric N deposition, fertilizer N application, agricultural N fixation, and N in net food and feed imports. Since N from sewage wastewater and animal feedlots are considered to be a part of food and feed consumptions which are included in the net food and feed imports, they are not estimated as new source of N. Atmospheric N deposition was estimated from REAS deposition data [31]. The amount of the applied N fertilizer was based on Hunan Statistic book. Agricultural N fixation was estimated from harvested area and N fixed rate per unit area for each crop type. In the Dongting Lake Basin, three main legume crops: peanuts, soybean and rice were included in the calculation. Net food and feed N imports were calculated as N consumed by human and livestock, subtracted by N production of livestock and crop. N consumptions were estimated by multiplying populations (rural and urban population for human, and 8 types of animal population for livestock) with their food N consumption per unit. N productions were estimated from 8 main animals and 14 main crops from the Statistic Book, and their N content value is derived from previous studies [32].

## Results

### Spatial distribution of TN in Dongting Lake

**Fig 2:**
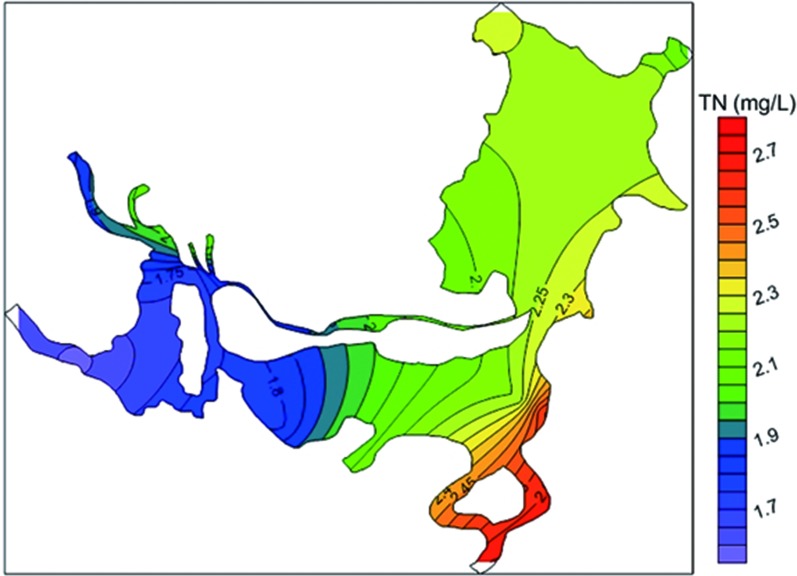
Spatial distribution of TN concentrations in Dongting Lake in 2014 (annual average).

The spatial distribution of TN concentrations in Dongting Lake in 2014 (annual average) is presented in Fig 2. Concentrations of TN ranged from 1.71–2.6 mg/L, with an average of 2.03 mg/L. The maximum value was found at S12 near the Xiangjiang inflow to SD, while the minimum value was found at S17 in WD. The spatial distribution of TN was more uniform in ED, while there were obvious concentration gradients in SD and WD. The nitrogen pollution levels in the upstream waters decreased in the following order: Xiangjiang > Zishui > three water inlets > Lishui > Yuanjiang.

N pollution was more serious in the southern area than in the western area. The TN concentrations decreased from the river inflows to the central area of the lake. The pollution was more serious in the upstream waters than in the lake water body (except in the Yuanjiang). If we take the Xiangjiang as an example, the average TN concentration in the Xiangjiang was 2.7 mg/L (Table 2), and the concentrations gradually decreased to 2.60 mg/L at S12 and further to 2.27 mg/L at S11 following the flow direction of the lake water body.

**Table 2:** TN concentrations in upstream inflows to Dongting Lake (mg/L).

	Xiangjiang	Zishui	Yuanjiang	Lishui	Yangtze River inlets
**Dry season average value ±standard deviation**	3.02±0.43	2.41±0.38	1.77±0.47	1.74±0.37	1.99±0.81
**Wet season average value ±standard deviation**	2.35±0.09	2.06±0.55	1.47±0.32	1.74±0.48	2.04±0.61
**Average**	2.7	2.13	1.58	1.79	1.97

### Seasonal variations of TN in Dongting Lake

**Fig 3:**
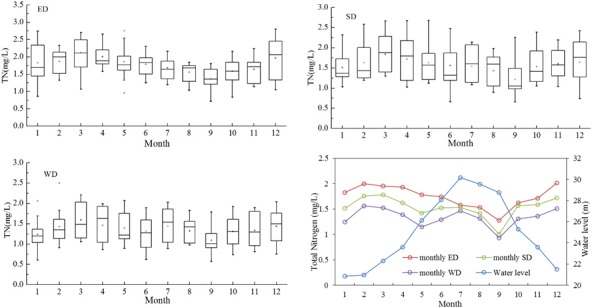
Seasonal variations in TN concentrations in different areas of Dongting Lake.

As shown in Fig 3, the monthly concentrations in ED, SD, and WD ranged from 1.28 to 2.01 mg/L, from 1.00 to 1.78 mg/L, and from 0.93 to 1.53 mg/L, respectively. The concentrations in the three lake areas were highest in March, and were lowest in September. The TN concentrations in the lake water showed clear seasonal variation, and exhibited a tendency to increase from the wet season to the normal season and then increased further in the dry season. The seasonal variations in the concentrations were most noticeable in SD, followed by ED, and were least noticeable in WD. Higher concentrations always occurred during the dry season in the upstream waters, while lower concentrations occurred during the wet season (Table 2).

Seasonal variations in the TN concentrations were negatively correlated with the water level (n = 12 for ED, SD, WD, r = −0.808, p<0.01 for ED, r = −0.649, p<0.05 for SD, r = −0.455, p>0.05 for WD). The concentrations were higher from December to March, when the water level was below 23 m, while the concentrations decreased when the water level was above 28 m from June to September.

### Inter-annual variations of TN in Dongting Lake

**Fig 4:**
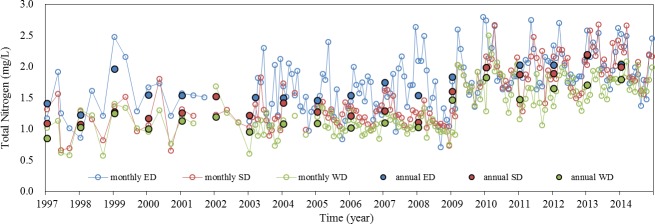
The monthly average concentrations of TN in Dongting Lake from 1997 to 2014

Inter-annual temporal variations of TN in ED, SD, and WD are shown in Fig 4. There was a significant increasing trend in TN concentrations (Kendall correlation coefficient: r = 0.57, P = 0 for ED; r = 0.67, P = 0 for SD; r = 0.63, P = 0 for WD), especially during the dry season. The inter-annual temporal changes of nitrogen can be divided into three stages. The first stage was a period of consecutive growth (1997–2002), during which the rates of increase for the different areas were in the order: ED > SD > WD. In the second period, the relatively stable period (2003–2008), the rates of increase in ED were greater than those in SD and WD. In the third period from 2009–2014, known as the dramatic increase period, the rates of increase in the TN concentrations were in the order: SD > WD > ED. The TN concentrations for the three stages are shown in Table 3 and S1 Table.

**Table 3:** TN concentrations in Dongting Lake during different time periods.

Time period		ED (mg/L)	SD (mg/L)	WD (mg/L)
**1997~2014**	Range	0.71(2008.9)~2.8(2009.12)	0.65(2000.9)~2.68(2013.5)	0.57(1998.9)~2.5(2010.2)
	average	1.77	1.56	1.35
	Increasing rate	43.97%	83.49%	108.14%
**1997~2002**	range	1.23~1.97	1.08~1.28	0.86~1.25
	average	1.51	1.17	1.09
**2003~2008**	range	1.46~1.75	1.11~1.42	1.02~1.09
	average	1.57	1.23	1.05
**2009~2014**	range	1.84~2.18	1.61~2.19	1.47~1.82
	average	2.02	1.94	1.68

**Fig 5:**
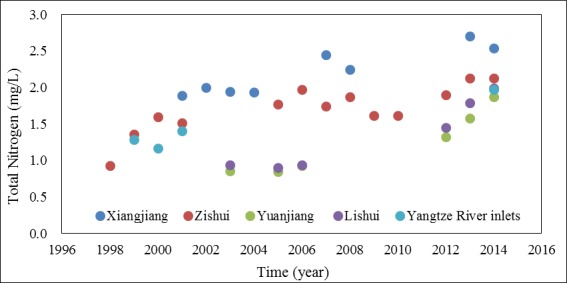
The annual average concentrations of TN in waters upstream of Dongting Lake from 1997 to 2014.

Concentrations of TN in all of the upstream waters showed increasing trends (Fig 5), and the rate of increase has been particularly evident in recent years, which is consistent with the trend in the lake water body. A comparison of the TN concentrations in the upstream waters with those in Dongting Lake showed that the TN pollution in the upstream waters was more serious than that in the lake water body. For example, the average TN concentration in the Xiangjiang (the largest river upstream of Dongting Lake) was 2.21±0.31 mg/L (from 2001 to 2014, n = 8), while it was 1.56±0.36 mg/L in SD, where the Xiangjiang enters the lake. Nitrate was the major form of TN both in Xiangjiang and SD, which accounted for 74.01% and 77.47% on average. Particulate nitrogen accounted for 16.04% and 14.34%, while ammonium only accounted for 9.95% and 8.2%.

### Variations in the TN composition in Dongting Lake

Dissolved inorganic nitrogen (DIN) represents the major N poor in the three lake areas, which accounted for between 63.5% and 99.8% of TN (average 87%); this portion can be directly taken up by phytoplankton. The content of the particulate nitrogen fraction was relatively low (0.28±0.06 mg/L). The seasonal variations in DIN and TN were similar. Nitrate was the major form of DIN (accounting for over 90%), and accounted for 91% in WD, 90.6% in SD, and 89.6% in ED. Ammonium accounted for only 9.6% of DIN, and accounted for 10.4% at ED, 9.4% at SD, and 9% at WD (9%).

**Fig 6:**
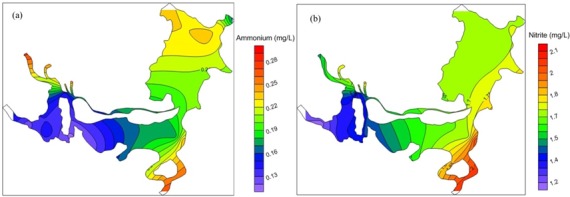
Spatial distribution of (a) ammonium and (b) nitrate in 2014.

The nitrate concentrations ranged from 0.52 to 2.48 mg/L, and had an average value of 1.58 mg/L (Table 4). Nitrate accounted for 77.83% of TN and was the largest fraction of TN. Nitrate concentrations were significantly positively correlated with TN concentrations (r = 0.811, n = 360, p<0.01), and showed similar seasonal and spatial variations (Fig 6).

**Table 4:** N composition in Dongting Lake in 2014 (the concentration is the average value±standard deviation).

	Dry season (mg/L)				Wet season (mg/L)			
concentration	ammonium	nitrite	DIN	TN	ammonium	nitrite	DIN	TN
**ED**	0.36±0.17	1.72±0.25	2.11±0.28	2.47±0.31	0.12±0.04	1.54±0.22	1.66±0.23	1.85±0.19
**SD**	0.30±0.21	1.76±0.31	2.02±0.49	2.34±0.65	0.11±0.06	1.55±0.26	1.66±0.26	1.94±0.39
**WD**	0.13±0.07	1.32±0.26	1.46±0.3	1.67±0.3	0.11±0.04	1.37±0.18	1.48±0.21	1.62±0.23

Ammonium concentrations ranged from 0.03 to 0.86 mg/L, and had an average value of 0.18 mg/L (Table 4). The concentrations during the dry season were higher than those during the wet season, especially in ED, where they were 3 times higher. There was no obvious seasonal difference in the concentrations in WD. Ammonium pollution was more serious in the northwestern area of ED and at the river inflows (Fig 6). Ammonium concentrations decreased from the river inflows to the central lake area.

## Discussion

### Factors that influence the spatial distribution of TN in Dongting Lake

Our results agree well with those of previous that reported a decrease in TN concentrations from the river inflows to the central lake area [33], and that the pollution was more serious in the southern area than in the western area [34]. We also observed large gradients in TN concentrations in both SD and WD, which indicated that the spatial distribution of TN was closely related to the entrance location and concentrations of the inflows from upstream. By combining the spatial distribution and the TN concentrations of the upstream waters, we found that the TN concentrations decreased in the following order: Xiangjiang (S1, 2.7 mg/L) > Zishui (S2, 2.13 mg/L) > three inlets (S5–S7, 1.97 mg/L) > Lishui (S4, 1.79 mg/L) > Yuanjiang (S3, 1.58 mg/L). Further, the maximum concentration occurred at S12 near the inflow of the Xiangjiang in SD, while the minimum concentration was observed at S17 near the inflow of the Yuanjiang in WD. The TN concentrations in WD (1.71 mg/L) were less than those in SD (2.19 mg/L). Also, the discharge amount decreased as follows: Xiangjiang (659.7×10^8^ m^3^) > Yuanjiang (640.6×10^8^ m^3^) > Songzi (417×10^8^ m^3^) > Ouchi (326×10^8^ m^3^) > Zishui (229.6×10^8^ m^3^) > Taiping (162×10^8^ m^3^) > Lishui (147.7×10^8^ m^3^). The water discharged in SD with higher concentrations from the Xiangjiang and the Zishui was diluted by the water discharged in WD with lower concentrations from the Yuanjiang and the Lishui. And there was a large gradient in the TN concentrations from SD to WD, which tended to increase from WD to SD. Previous studies have demonstrated that inflows from upstream were the main pollutant pathway into Dongting Lake, accounting for over 80% of the total load [35]. Therefore, the spatial position of the upstream waters and the seasonal variations in their water quantity and quality were responsible for the spatial distribution of nitrogen in the lake. Some studies of Taihu Lake have reported similar findings [8, 36].

**Fig 7:**
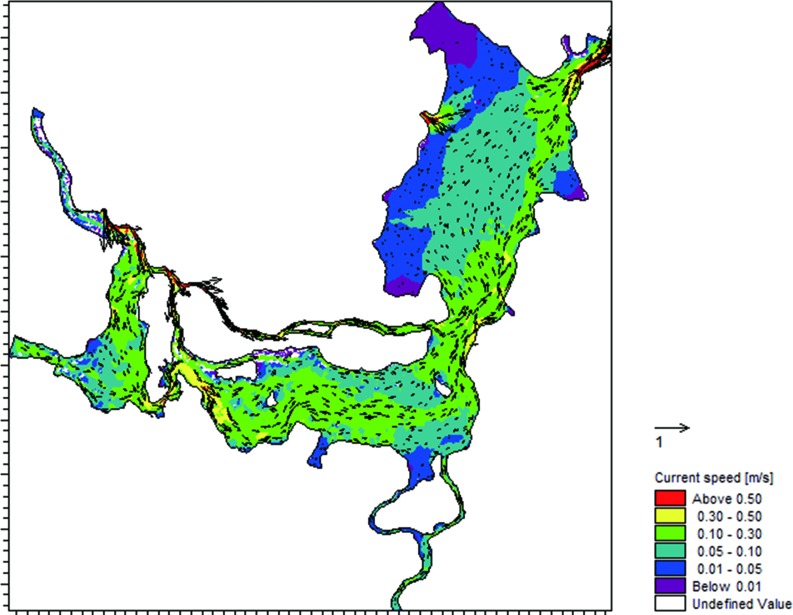
Velocity and direction of flow in Dongting Lake.

Although few of the upstream rivers flowed into ED, the TN concentrations in ED were maintained at a high level and were between the concentrations at SD and WD. This phenomenon is mainly related to the flow direction and the flow velocity in the lake water body. The flow direction of water in Dongting Lake is from west to south then to east (Fig 7). That is, the water from WD and SD joined and mixed at the entrance of ED, and then entered ED. Thus, nutrients were transported with water flow from WD to SD to ED, which led to the TN concentrations in ED were between those at SD and WD. Meanwhile, as shown in Fig 7, the flow velocity in ED (0.036 m/s) was much lower than those in WD (0.136 m/s) and SD (0.162 m/s).

As a result, this kind of change not only weakened the water exchange and reduced the self-purification capacity, but also led to easier deposition of pollutants. N release from sediments represents a secondary source of pollution and can cause water quality deterioration [37]. Wang et al. [34] pointed out that the TN concentrations were higher in the sediments in ED (1,513.43 mg/kg) than in WD (1,173.14 mg/kg) and SD (1,262.76 mg/kg), which indicates a higher risk of secondary nitrogen release. In a word, the flow direction and velocity were responsible for the pollutant accumulation at ED. Shen et al. [38] also mentioned that the low flow velocity was an important contributor to the high concentrations in this region.

### Factors influencing seasonal variations in TN in Dongting Lake

As mentioned in section Inter-annual variations of TN in Dongting Lake, TN concentrations exhibited apparent seasonal variations over the past two decades and increased from the wet season to the normal season to the dry season. The concentrations of the upstream waters were also much higher in the dry season than in the wet season. Statistical analysis indicated that the TN concentrations in the Xiangjiang (S1) were positively and significantly correlated with those of ED (r = 0.744, p<0.01) and SD (r = 0.755, p<0.01), and that the TN concentrations of the Zishui (S2) were significantly and positively correlated with ED (r = 0.680, p<0.01). These relationships indicate that seasonal variations in TN in the upstream waters and the spatial positions of the inflows were the major controls on seasonal changes in nitrogen in ED and SD. The water quality of WD however was influenced by the combination of five upstream rivers (Yuanjiang, Lishui, Songzi, Taiping and Ouchi).

As a seasonally regulated lake, Dongting Lake demonstrated significant variation in hydrodynamic conditions during the dry and wet seasons [29]. As a result, the water quality was not only influenced by the pollution load that entered the lake, but was also closely related to its unique hydrological regime [39]. The water storage capacity in the wet season was about 17–31 times that in the dry season, and the increase in the storage capacity during the wet season contributed to the dilution of pollutants [40]. In contrast, during the decreased water storage in the dry season, the lake beaches were exposed, the water exchange capacity declined, and the pollutant transmission rate decreased [42]. This led to an increase in pollutant retention rate, which in turn exacerbated the severity of the TN pollution in the dry season. Ni et al. [12] also suggested that the hydrodynamic regime was the most important factor controlling water quality in Poyang Lake: the higher the water level, the better the water quality, and vice versa. However, Wang et al. [34] reported that the TN concentrations during the wet season were higher than those during the dry season. Their results were different because of their chosen monitoring frequency; they only sampled twice, in January and June 2012, therefore the data were limited and not representative of the whole year.

### Factors influencing annual variations in TN in Dongting Lake

The Dongting Lake basin is one of most densely populated regions in China, and the population density (407 individuals/km^2^) is 3.9 times that of the whole country [25]. It is also an important agricultural production base, and its grain production accounted for 5.78% of the total grain production in China in 2014 [25]. NANI from human activities in Dongting Lake Basin (23257.9 kg N km^-2^,2014) was currently five times the China average, and the fertilizer N application was the main source (52.55%). Therefore, if natural conditions are excluded, changes in the TN concentrations are closely related to human activities in the basin. Historical changes in TN concentrations can be divided into three stages.

**Fig 8:**
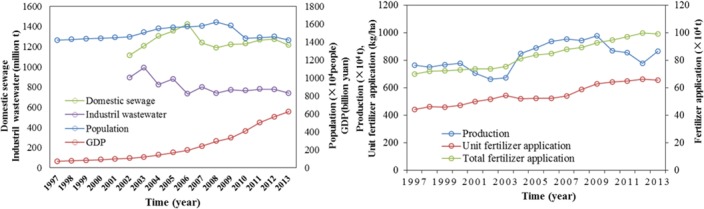
Historical changes in NANI and relationship between fertilizer N inputs and TN concentrations in upstreams.

The first stage can be called the consecutive growth stage (1997–2002). During this period, economic activities began to intensify in China [12]. The regional GDP of Dongting Lake basin increased by 45.71% and the population density increased by 3.7%, reaching 383 individuals/km^2^. The positive linear relationship between population density and NANI (r^2^ = 0.835, p<0.01) indicated that the rising population intensity enhanced the loads of nitrogen from domestic and agricultural sewage and led to increase in NANI from 19307.9 kg N km^-2^ to 20716.7 kg N km^-2^ (Fig 8). We also examined the relationship of TN concentration in the lake and NANI inputs to the basin, and found positive linear relationship between them (r^2^ = 0.75, p<0.01). Accordingly, TN concentration in Dongting Lake which received N input from the lake drainage area has increased by 21.69%.

**Fig 9:**
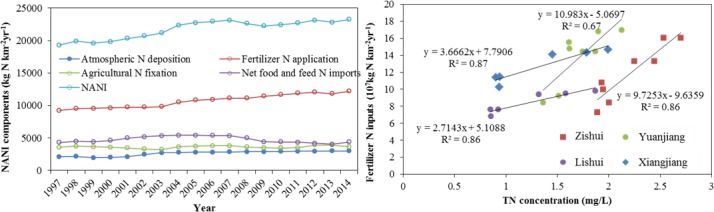
Historical changes in selected social, industrial, and agricultural parameters in Dongting Lake.

The second stage can be termed the relatively stable stage (2003–2008). The local government implemented a series of measures to halt the trend of water quality deterioration [19, 42], such as controls on the sources of heavy industrial pollution and concentrated treatment of urban sewage. Therefore, discharges of industrial wastewater decreased by 50.87 million t (about 5.12%) annually and the wastewater treatment rate increased from 26.81% to 52.05% from 2003 to 2008 (Fig 9). These measures effectively reduced the external load of nitrogen, and so, to some degree, restricted the increases in TN concentrations. During this period the proportion of industrial and domestic pollution decreased, but the proportion of non-point source pollution began to increase [19].

The third stage can be called the dramatic increase stage (2009–2014). The TN concentrations of ED continued to increase, and dramatic increases were also observed in SD and WD (Fig 3), where the concentrations increased by 57.99% and 60.41% relative to the second stage. These dramatic concentrations exacerbated the eutrophication status, and algal blooms appeared [33]. Dongting Lake was experiencing the critical transition from a macrophytic lake with a high self-purification capacity to an algal lake with a high nutrient load. Although the discharges of industrial wastewater continued to decrease, and the level of wastewater treatment improved further, these changes were not translated into improvements in water quality. According to the structure of NANI, fertilizer N application was becoming increasingly significant which accounting for 52.55% of NANI in 2014, while the other components of NANI were of minor importance (Fig 8). This implies that N loading from fertilizer N application was an important source of N input in Dongting Lake basin.

Dongting Lake basin is an important agricultural production base in China, so farmers increased their chemical fertilizer applications, hoping to increase food production to meet the needs of the rapidly growing population. During the past two decades, the amount of chemical fertilizer consumed by unit area increased by 47.84%, and reached 655.22 kg/ha in 2014, which was even greater than the amount used in areas of serious agricultural pollution, such as Erhai (230 kg/ha) [12]. However, the growth rate in the grain production by unit area (13.02%) was much less than the growth rate in chemical fertilizer applications (47.84%), and the amount of grain produced began to decrease after 2009 (Fig 9). These results indicate that the use efficiency of the chemical fertilizer decreased gradually over a number of years, so that more and more chemical fertilizer accumulated in the soil. Specifically, the excessive application of N fertilizer was much higher than that of P and K fertilizer, and the soil loss rate of N fertilizer to the water was as high as 68%. In addition, a survey showed that the total area at risk of soil erosion (569,599 ha^2^) covered 12.54% of the total land area of the Dongting Lake basin [25], resulting in a huge loss of N from land to the lake [43]. Several studies have indicated that excessive applications of chemical fertilizer are the main source of nutrient inputs [44, 45]; chemical fertilizers account for 80% of the total nutrient inputs to Dongting Lake [46] and overuse of chemical fertilizers has resulted in N accumulation [19, 43]. The significant positively relationship between fertilizer N application inputs and TN concentrations for each upstream (Fig 8) proved that fertilizer N inputs were the main source of N input of Dongting Lake and the upstream N concentrations were highly correlated to fertilizer N inputs. As mentioned in section 4.1, inflows from upstream were the main pollutant pathway into Dongting Lake. Thus, the excessive nitrogen pollutants transported from upstream to lake which led to the increase of TN concentration of lake water. In addition, fertilizer N applications were also significantly and positively correlated with TN concentrations of lake water (r = 0.848, n = 18, p<0.01). These implied that the increasing fertilizer N input was the main reason in the increase in TN concentrations in Dongting Lake. Currently, the combined influence of decreasing rainfall and changing interactions between the Yangtze River and the lake caused by the Three Gorges Dam impoundment means that the amount of water entering Dongting Lake has decreased over recent years [29]. Compare with average annual discharge amount, the river discharge of the three inlets from the Yangtze River (TIYR) and four upstream rivers (FUR) has decreased 28.09% (from 687×10^8^m^3^ to 494×10^8^m^3^) and 7.13% (from 1724×10^8^m^3^ to 1601×10^8^m^3^) in recent years. But the TN concentrations of the upstream waters of Dongting Lake have increased gradually, which has increased by 128.9% for FUR and 34.67% for TIYR. In particular, compared to the water discharged from FUR with higher TN concentration (2.13 mg/L), the water discharged from TIYR with lower TN concentration (1.97 mg/L) has decreased more significant and rapid [27, 41]. These meant that the dilution effects of the upstream waters for the lake body weakened and the lake body continued to receive high TN concentration inputs, which led to the increase of TN concentrations of lake body to a certain extent.

**Fig 10:**
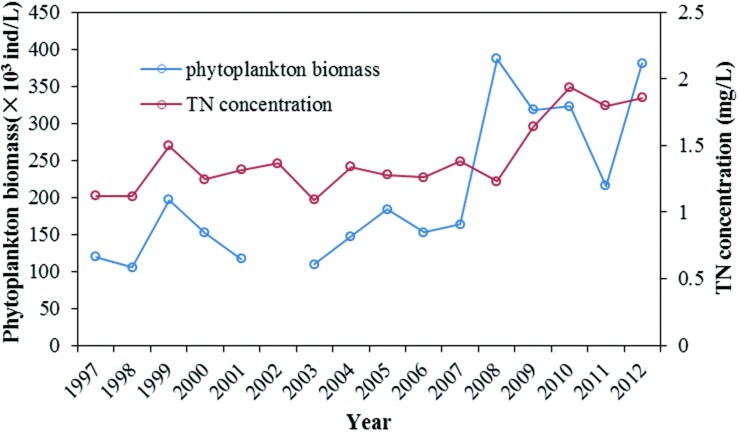
Historical changes in phytoplankton biomasses and TN concentrations in Dongting Lake.

The increase of nitrogen concentration in Dongting Lake provided the material foundation for the growth of phytoplankton. Fig 10 shows an overview of the phytoplankton biomass, with corresponding TN concentrations in Dongting Lake during the past two decades. During the past two decades, the phytoplankton biomass increased gradually (Fig 10). In addition, phytoplankton biomass is significantly correlated with TN (r = 0.666, n = 17, p<0.01). This implies that the excessive TN concentration was the most important factor in the increase in the phytoplankton biomass in Dongting Lake. As the TN concentrations increase gradually, the eutrophication risk in Dongting Lake will continue to rise.

## Conclusion

Results from our study have demonstrated that TN concentrations decreased from the river inflows to the central lake area, and that TN pollution was more serious in the southern area than in the western area. Concentrations of TN varied seasonally, and were significantly higher during the dry season than during the wet season. The spatial and temporal variations in the TN concentrations were attributed to the positions of the upstream inflows to the lake and seasonal variations in the quantity and quality of their inputs. Spatio-temporal variations in hydrodynamic conditions (flow velocity, flow direction, etc.) in the lake water body were the second most important factor.

The increased concentrations in Dongting Lake over the past two decades reflect the combined influence of industrial wastewater, domestic sewage, agriculture chemical fertilizer loss, and reduction in water discharges. The annual changes in nitrogen can be divided into three stages: consecutive growth (1997–2002), relative stability (2003–2008), and dramatic increase (2009–2014). Increasing domestic and industrial wastewater discharges, the implementation of pollution control policies, excessive applications and low uptake of chemical fertilizer, and decreased discharges, were the main drivers of change in the TN concentrations in the different stages.

Losses of agricultural chemical fertilizers are currently the main source of TN pollution in the Dongting Lake basin, and the main pollutant pathway is the influx from the upstream waters. As regards water management, the local government should endeavor to control the pollutant influxes from the upstream waters rather than from areas surrounding the lake, giving priority (in decreasing order) to the Xiangjiang, Zishui, the three water inlets connected with the Yangtze River, the Lishui, and the Yuanjiang. Non-point source pollution control rather than point source pollution should be enhanced in the future, and, in particular, losses of agricultural chemical fertilizer. To resolve these problems, we recommend that attention is directed towards agricultural improvements, and efforts should be made to replace chemical fertilizers with organic and biological fertilizers to reduce nutrient losses from farmland.

## Supporting information

S1 Table Dongting Lake Data. (XLSX)
